# Assessing the robustness of radiomics/deep learning approach in the identification of efficacy of anti–PD-1 treatment in advanced or metastatic non-small cell lung carcinoma patients

**DOI:** 10.3389/fonc.2022.952749

**Published:** 2022-08-05

**Authors:** Qianqian Ren, Fu Xiong, Peng Zhu, Xiaona Chang, Guobin Wang, Nan He, Qianna Jin

**Affiliations:** ^1^ Department of Radiology, Union Hospital, Tongji Medical College, Huazhong University of Science and Technology, Wuhan, China; ^2^ Hubei Province Key Laboratory of Molecular Imaging, Tongji Medical College, Huazhong University of Science and Technology, Wuhan, China; ^3^ Department of Hepatobiliary Surgery, Wuhan No.1 Hospital, Wuhan, China; ^4^ Department of Pathology, Union Hospital, Tongji Medical College, Huazhong University of Science and Technology, Wuhan, China; ^5^ Department of Gastrointestinal Surgery, Union Hospital, Tongji Medical College, Huazhong University of Science and Technology, Wuhan, China; ^6^ Cancer Center, Department of Gastrointestinal Surgery, Union Hospital, Tongji Medical College, Huazhong University of Science and Technology, Wuhan, China

**Keywords:** NSCLC, radiomics, deep learning, robustness, immunotherapy

## Abstract

Administration of anti–PD-1 is now a standard therapy in advanced non-small cell lung carcinoma (NSCLC) patients. The clinical application of biomarkers reflecting tumor immune microenvironment is hurdled by the invasiveness of obtaining tissues despite its importance in immunotherapy. This study aimed to develop a robust and non-invasive radiomics/deep learning machine biomarker for predicting the response to immunotherapy in NSCLC patients. Radiomics/deep learning features were exacted from computed tomography (CT) images of NSCLC patients treated with Nivolumab or Pembrolizumab. The robustness of radiomics/deep learning features was assessed against various perturbations, then robust features were selected based on the Intraclass Correlation Coefficient (ICC). Radiomics/deep learning machine-learning classifiers were constructed by combining seven feature exactors, 13 feature selection methods, and 12 classifiers. The optimal model was selected using the mean area under the curve (AUC) and relative standard deviation (RSD). The consistency of image features against various perturbations was high (the range of median ICC: 0.78–0.97), but the consistency was poor in test–retest testing (the range of median ICC: 0.42–0.67). The optimal model, InceptionV3_RELF_Nearest Neighbors classifiers, had the highest prediction efficacy (AUC: 0.96 and RSD: 0.50) for anti–PD-1/PD-L1 treatment. Accuracy (ACC), sensitivity, specificity, precision, and F1 score were 95.24%, 95.00%, 95.50%, 91.67%, and 95.30%, respectively. For successful model robustification, tailoring perturbations for robustness testing to the target dataset is key. Robust radiomics/deep learning features, when paired with machine-learning methodologies, will work on the exactness and the repeatability of anticipating immunotherapy adequacy.

## Introduction

The introduction of programmed death 1 receptor (PD-1)/programmed death ligand 1 (PD-L1) blocking antibodies and targeted agents have substantially changed the therapeutic strategies for advanced lung cancer. In the setting of pre-treated patients with advanced non-small cell lung carcinoma (NSCLC), Nivolumab and Pembrolizumab monotherapy showed significantly better overall survival (OS), compared with traditional chemotherapy (1–3). Several predictive biomarkers based on cellular phenotypes, immunohistochemical, mutational tests, and expression-based approaches have been proposed to predict response to immune checkpoint inhibition. However, the predictive power of these methods was far from perfect. For example, only 44.8% of PD-L1–positive NSCLCs were responsive to Pembrolizumab in a first-line setting (4). Furthermore, it is difficult to identify the current status of immune profiles from an archival sample due to the dynamical evolution of the immune-escape mechanism during anti-cancer treatment (5, 6). Therefore, non-invasive methods, understanding the dynamics of the tumors in clinical practice, and assessing the immune landscape of tumors are critical.

Radiomics/deep learning (DL) image features are becoming a promising non-invasive method to obtain quantitative measurements for tumor classification and assessment for therapy response in oncological research (7–9). An imaging biomarker should be reproducible, robust, and accurate. However, image features are susceptible to several factors, such as imaging protocol variability, different vendors, image reconstruction processes, inter-rater tumor segmentation variability, patient motion artifact, overall image quality, and tumor phenotype (10–13). Ideally, only features that are robust to these variations would be incorporated into a predictive model for good generalizability (14).

We hypothesized that the combination of machine learning (ML) technologies and high-dimensional radiomics/DL features would facilitate the prediction of immunotherapy efficacy. Therefore, we investigated the robustness of radiomics/DL features against different perturbations and then determined the optimal model by combining feature extractors, feature selectors, and ML classifiers.

## Materials and methods

### Whuh (Wuhan Union Hospital) data

The medical records of patients with advanced NSCLC who had received Nivolumab (3 mg/kg every 2 weeks) or Pembrolizumab (200 mg every 3 weeks) monotherapy between January 2019 and January 2021 were retrospectively reviewed at Union Hospital, Tongji Medical College, Huazhong University of Science and Technology. Treatments were provided until disease progression, intolerable side effects, or consent to the withdrawal. The retrospective study was approved by the Ethics Committee of Union Hospital, which also waived the written informed consent, because the data were analyzed anonymously.

Patient inclusion criteria were (1) pathologically confirmed NSCLC, (2) enhanced computed tomography (CT) performed fewer than 15 days before treatment, and (3) availability of clinical data. The exclusion criteria were (1) missing or low-quality treatment CT, (2) suffering from other tumor diseases at the same time, (3) combining other treatments while using immunotherapy, (4) Patients with no measurable lesion by Immune-Modified Response Evaluation Criteria In Solid Tumors (imRECIST) or no available response evaluation (15). Tumor response to Nivolumab or Pembrolizumab monotherapy was objectively assessed by experienced radiologists (QQ. R, QN. J) using imRECIST in the third month. The details regarding the response assessment were described in the supplemental. The data pertaining to demographics, smoking history, histology type, TNM stage, and molecular testing and the number of prior lines of therapy were extracted from electronic medical records (**Table 1**).

**Table 1:** Demographic and clinical characteristics of patient populations.

Characteristic	Responsive to Immunotherapy (n=124)	Unresponsive to Immunotherapy (n=33)	*p*
Age (mean±SD [years])	59.69±8.19	58.42±8.89	
Gender			0.336
Male	103	25	
Female	21	8	
Smoking History			0.140
Yes	89	19	
No	35	14	
HistoType			0.108
A	85	19	
S	36	10	
U	2	3	
AS	1	1	
Clinical Stage			0.378
IIIB	25	9	
IV	99	24	
The expression of EGFR			0.267
Positive	16	1	
Negative	28	8	
Unknown	80	24	
The expression of ALK			0.556
Positive	1	1	
Negative	35	8	
Unknown	88	24	
The level of PD-Ll			0.235
High	36	6	
Low	20	9	
Unknown	68	18	
Chemotherapy			0.194
1 course	21	10	
2 courses	43	8	
3 courses	60	15	

U, undifferentiated large cell carcinoma; A, adenocarcinoma; S, squamous cell carcinoma; AS, adenosquamous carcinoma.

### Test–retest cohorts

The test–retest cohort with 31 NSCLC patients was available from the Cancer Imaging Archive (16, 17). Images in the test–retest cohort using the same scanner and acquisition protocol were acquired every 15 min. Informed consent was waived.

### Computed tomography acquisition and segmentation

CT scans were acquired using a multi-slice spiral CT system (Philips Healthcare, General Electric Health Care, and Siemens Healthcare) with a tube voltage of 100–120 kVp, slice thickness (spacing) of 1–5mm, and in-plane resolution of 0.75 mm × 0.75 mm. All scans were acquired using the facilities’ CT chest protocol and standard image reconstruction.

### Pre-processing and tumor segmentation

The tumor regions of interest (ROIs), which corresponded to the biggest target lesion, were manually performed using three-dimensional Slicer software, which was based on a consensus reached by two experienced radiologists (one with 5 years of experience, another with 10 years of experience). For those cases with a blurred edge around the lesion, the maximum range was drawn and regarded as the border. Large vessels, adjacent organs, and air cavities were excluded. On contrast-enhanced CT, difficult-to-identify lesions were labeled with reference to the corresponding nuclear positron emission tomography (PET) image (some patients had PET scans) or with the permission of two physicians. The two readers repeated the same procedures 2 weeks later and any disagreement was resolved through consultation.

### Feature extraction

To be consistent with DL features, three consecutive slices with the maximum cross-sectional area of the tumor lesion were selected. Radiomic feature calculations were automatically done using the PyRadiomics package implemented in Python (18). Radiomics features with or without wavelet filtration included three groups: (1) first-order statistics, (2) shape features, and (3) second-order features: gray-level co-occurrence matrix (GLCM), gray-level size zone matrix (GLSZM), gray-level run-length matrix (GLRLM), neighborhood gray-tone difference matrix (NGTDM), and gray-level dependence matrix (GLDM) features (18).

ImageNet, which has numerous object categories and manually annotated training photos, was used to pre-train InceptionResnetV2, InceptionV3, Resnet50, VGG16, VGG19, and Xception (19). The six pre-trained CNNs were used as an arbitrary feature extractor while executing DL feature extraction, allowing the input picture to propagate forward, halting at the penultimate layer, and using the outputs of that layer as our features. We used global max pooling to extract the feature map’s maximum value before converting it to its original value.

### Image normalization

An image interpolation procedure was needed to standardize the images after CT image acquisition and segmentation. The image brightness was adjusted through the adaptive window level. The histogram equalization method was applied to CT images to get better visualization. The size of the three axial slices was adjusted to 224 mm × 224 mm, consistent with the input layer size of the pre-trained CNN models. The Gaussian filter was used to remove noise in images since CT images were mainly affected by quantum noise, which would be caused by the variability of the electron density of tissue voxels, and represented by random Gaussian process statistics (20).

### Robust features for test–retest imaging and image perturbations

We tested feature robustness against various perturbations in Whuh data, then feature robustness was verified in the test–retest cohort.

According to the imaging guidelines (21) and the radiologist’s visual inspection, we defined the expected perturbations in a multicenter setting.

**Figure 1:**
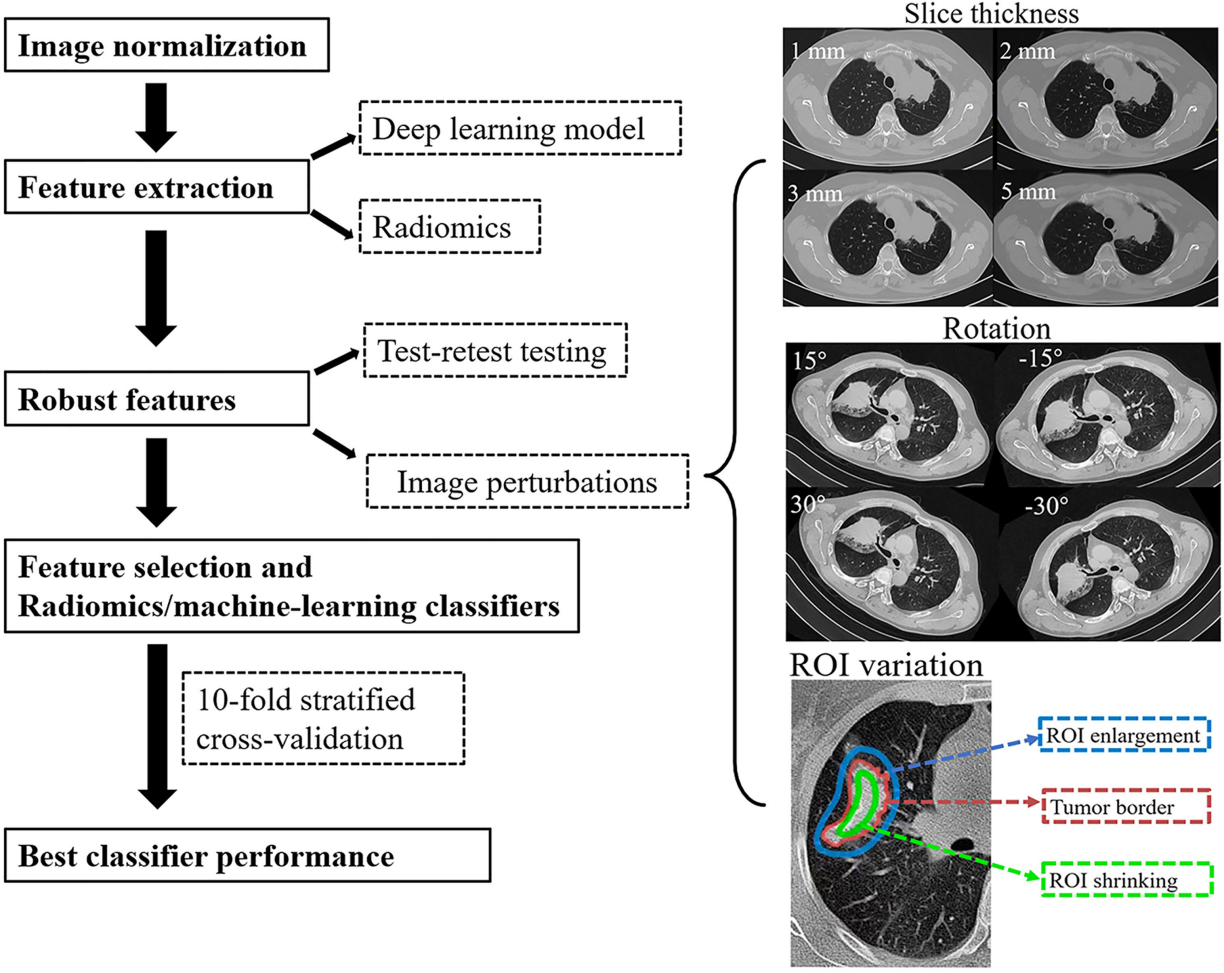
The study flowchart. After pre-processing and tumor segmentation, the images were artificially perturbed. Robust features were evaluated by machine learning (ML) models.

(1) Axial slice spacing (S): CT images were reconstructed contiguously at 1, 2, 3, and 5 mm section thicknesses.
(2) Rotation (R): The depicted tumor rotation would be affected by the patient’s position. Therefore, we generated a set angle θ [−30°, −15°, 15° 30°] and rotated the image, and segmented tumor in the axial (*x*, *y*) plane.
(3) ROI variation (Seg): The depicted tumor edge might be affected by the patient’s respiratory motion artifact and the variability of intra- and inter-observer ROI segmentation. Therefore, ROI enlargement and shrinking were considered (enlargement and shrinking were shown in **Figure 1**) (14, 22).

Robust features evaluation: ICC (2,1) for each feature was calculated and only those that reach the cutoff (ICC > 0.75) for all tested perturbations were entered following the feature selection and modeling process. Raw feature vectors were further standardized by being centered to the mean and scaled to unit variance. Features with zero median absolute deviation (MAD), regarded as nonpredictive features, were further removed.

### Feature selectors and machine learning methods

The feature selection methods included chi-square score (CHSQ), ReliefF(RELF), mutual information maximization (MIM), Fischer Score (FSCR), mutual information feature selection (MIFS), Gini index (GINI), interaction capping (ICAP), joint mutual information (JMI), conditional infomax feature extraction (CIFE), conditional mutual information maximization (CMIM), double input symmetric relevance (DISR), minimum redundancy maximum relevance (MRMR), and test score (TSCR).

The 12 ML classifiers included logistic regression, k-nearest neighbors, quadratic discriminant analysis (QDA), Support Vector Classifiers (SVCs) with linear and radial basis function (RBF) kernels, XGBoost, multilayer perceptrons, Gaussian processes, decision trees, naive Bayes, random forests, and AdaBoost. These classifiers were all imported from a Python (version 3.6.4) ML library named scikit-learn (version 19.0) (23). Further details about the feature selection methods were in **Supplementary S2**, and the parameter settings and tuning range of ML classifiers were detailed in the **Supplementary Materials**.

### Machine learning and model performance evaluation

Seven feature extractors, 13 feature selectors, and 12 classifiers were combined, then 1,092 (7 × 13 × 12 = 1092) ML models were generated. The nomenclature of each model combined the feature exactor, the names of the feature selector, and the classification method. For example, Rnest50_ RELF _ nearest neighbors was a model trained by a k-nearest neighbors classifier with features selected by the ReliefF and extracted from Rnest50.

Each of the 1,092 models was trained during the 10-fold stratified cross-validation using the StratifiedKFold iterator in scikit-learn, which is a variation of kfold cross-validation that ensured each set contained approximately the same percentage of samples of each target class as the whole training dataset. Synthetic minority over-sampling technique was adopted to handle the imbalanced data.

The best performing model was selected based on AUC and relative standard deviation (RSD). RSD was defined as the ratio between the standard deviation and mean of the 10-fold cross-validated AUC values: RSD = (sdAUC/mean AUC) ×100. The lower the RSD value, the higher the stability of the predicting model. The model with the highest AUC value and the lowest RSD was considered the best performing model. The performance of the best performing model was further measured by accuracy (ACC), sensitivity, specificity, F1 score, and precision.

### Statistical analysis

Continuous variables were presented by using median with mean + SD and the statistic difference was compared by Wilcoxon signed-rank test. For differences in categorical variables, Fisher’s exact test was adopted, and the results were shown as the number of events followed by relative frequencies (%). A two-sided *p* < 0.05 was used as the criterion to indicate a statistically significant difference.

## Results

The study flowchart was presented in **Figure 1**.

### Patient characteristics

Of 157 patients with advanced NSCLC (128 men, 29 women), 109 patients underwent nivolumab monotherapy and 48 underwent pembrolizumab monotherapy during the study period. The median age was 59 (range: 29–78) years. One hundred four (66%) were diagnosed as having adenocarcinoma, 46 (29.3%) were squamous cell carcinoma, five (3.2%) were undifferentiated large cell carcinoma, and two (1.3%) were adenosquamous carcinoma. Mutations in epidermal growth factor receptors were present in 17 patients (10.8%). Thirty-one patients (19.7%) had received one course of chemotherapy, 51 patients (32.5%) had received two courses, and 75 patients (47.8%) had received three or more courses. The expression of PD-L1 was abundant (tumor proportion score [TPS] ≥ 50%) in 42 patients (26.8%), at low levels (1% ≤ TPS < 50%) in 29 patients (18.5%), and unknown in the remaining 86 (54.8%). According to Response Evaluation Criteria in Solid Tumors, version 1.1, after anti–PD-1 immunotherapy, 65 patients (41.4%) had a partial response, 59 patients (37.6%) had stable disease, and 33 patients (21.0%) had progressive disease (**Table 1**).

### Feature robustness

**Figure 2:**
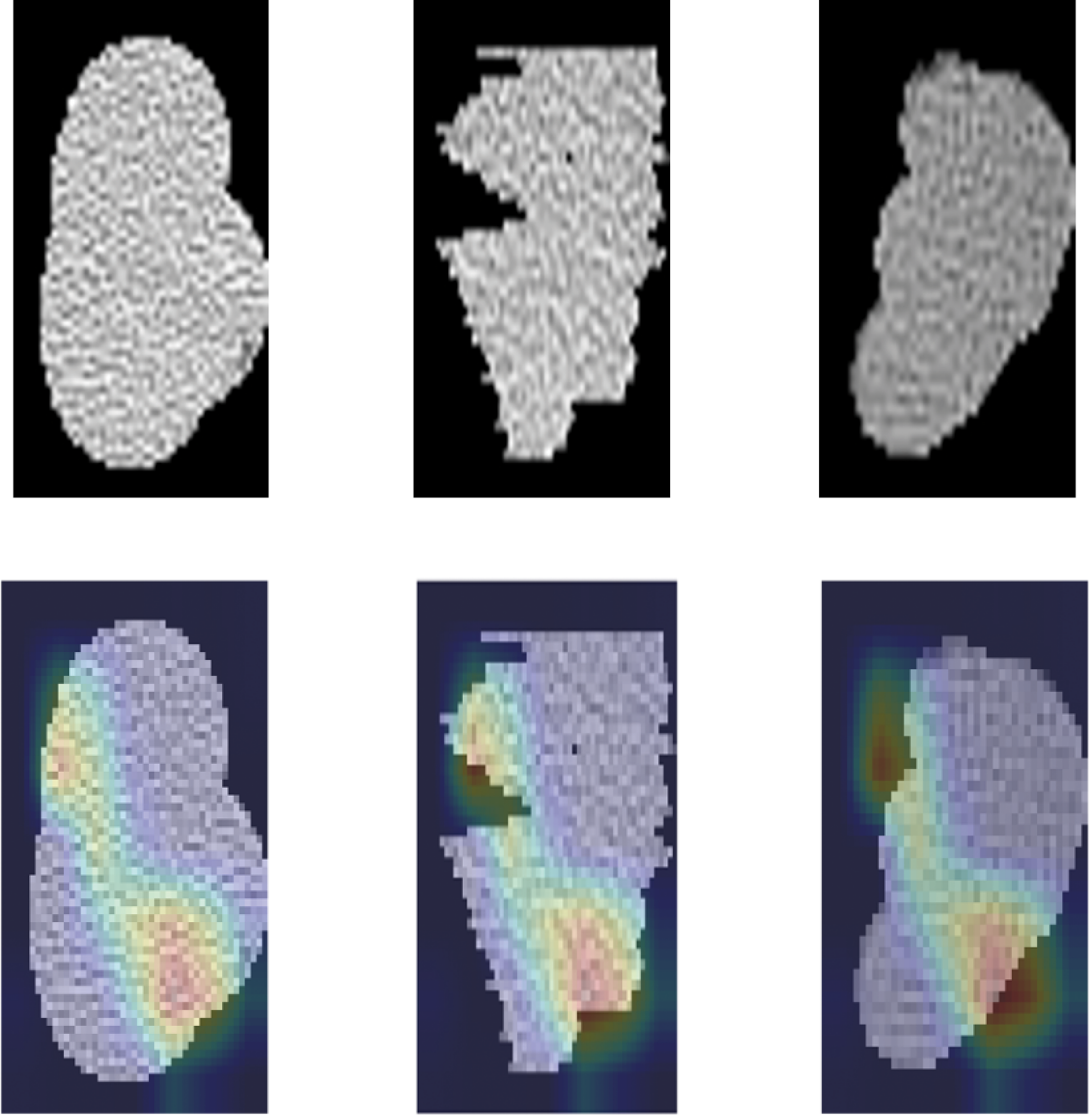
The heatmap of features generated from InceptionV3 for representative patients.

One hundred seven original features and 744 wavelet features were extracted concerning radiomics features. Radiomics features included 14 shape parameters, 162 first-order parameters, 216 GLCM parameters, 144 GLRLM parameters, 144 GLSZM parameters, 126 GLDM features, and 45 NGTDM parameters. The number of features for DL models was InceptionResNetV2 1536, InceptionV3 2048, Xception 2048, and Resnet50 2048, VGG16 512 and VGG19 512. The representative feature heatmaps of features generated from InceptionV3 were presented in **Figure 2**.

**Figure 3:**
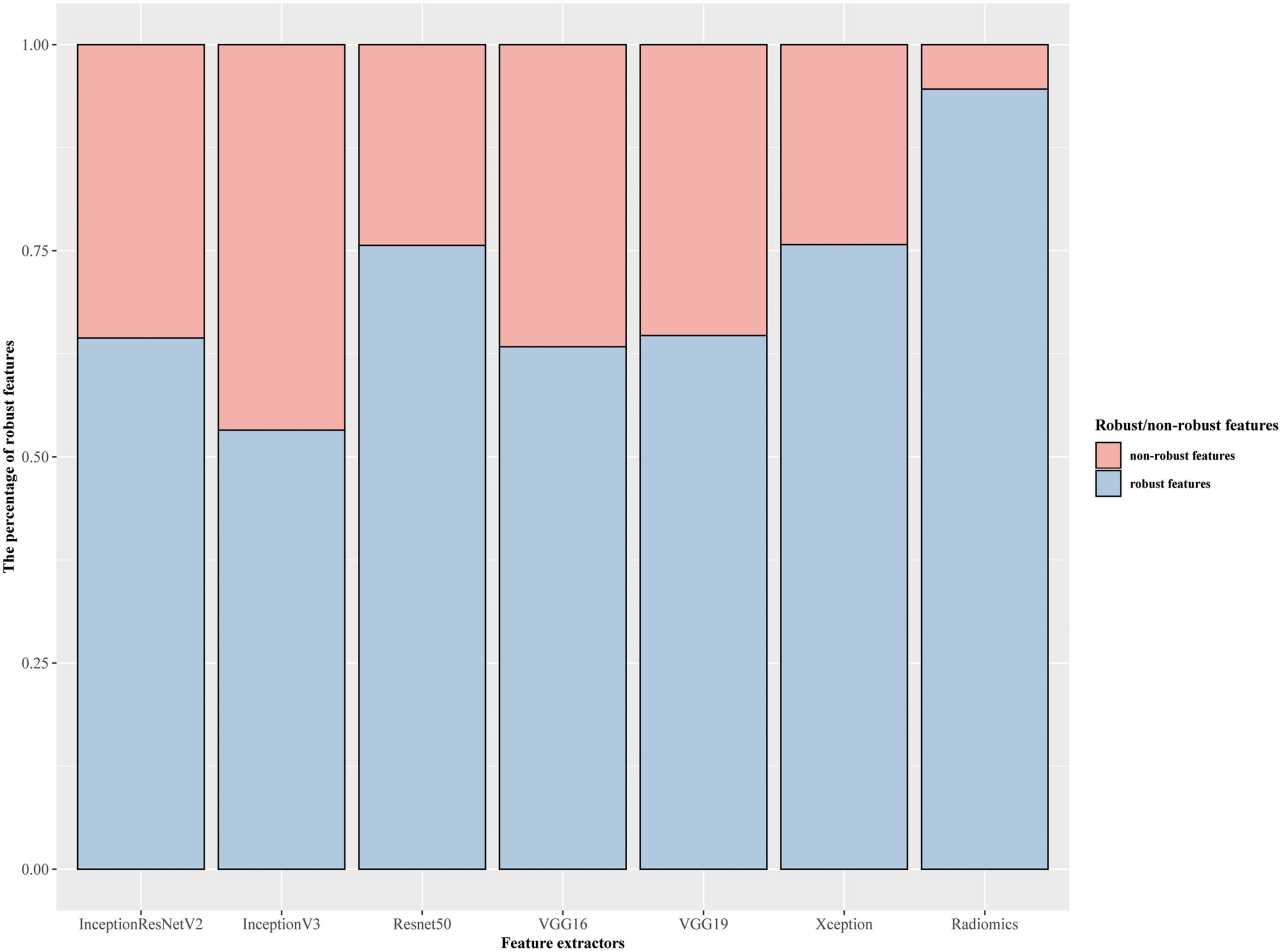
Overall percentage of robust features against image perturbations.

In DL and radiomics features, ICCs ranging from 0.80 to 0.90 demonstrated favorable feature reproducibility for S (axial slice spacing). The features from InceptionResnetV2 and InceptionV3 were robust against R(rotation) but have a lower agreement if the ROI changed. For features from Resnet50 and Xception, robustness against S and Seg (ROI variation) were comparable. The features from VGG16 and VGG19 were robust against Seg but had a lower ICC for R. Radiomics features were robust against each perturbation, especially against Seg. The percentage of robust features against all perturbations for each feature extractor was presented in **Figure 3** (The performance of each feature extractor against each image perturbation was reported in **Supplementary Table 1** with median and the interquartile range (IQR)). The number of robust features for different ICC threshold settings was reported in the **Supplementary Material Figure 1**.

Compared with the consistency test for various perturbations, the repeatability in the test–retest group was much worse. The ICC of the best radiomic features in the above robustness testing was 0.6 in the test–retest group. The performance of each feature extractor regarding the test_retesting images was reported in **Supplementary Table 2** with median and IQR.

We then reduced the number of features by removing features with zero MAD across the two cohorts. With the ICC threshold set to 0.75, the numbers of features remaining after robustness testing were radiomics 233, InceptionResNetV2 25, InceptionV3 74, Resnet50 109, VGG16 30, VGG19 73, and Xception 50. These features were first screened by the 13 feature selectors mentioned, and then the best combination was further screened by the wrapper feature selection method based on the recursive feature addition algorithm.

### Feature selection and machine learning models

**Figure 4:**
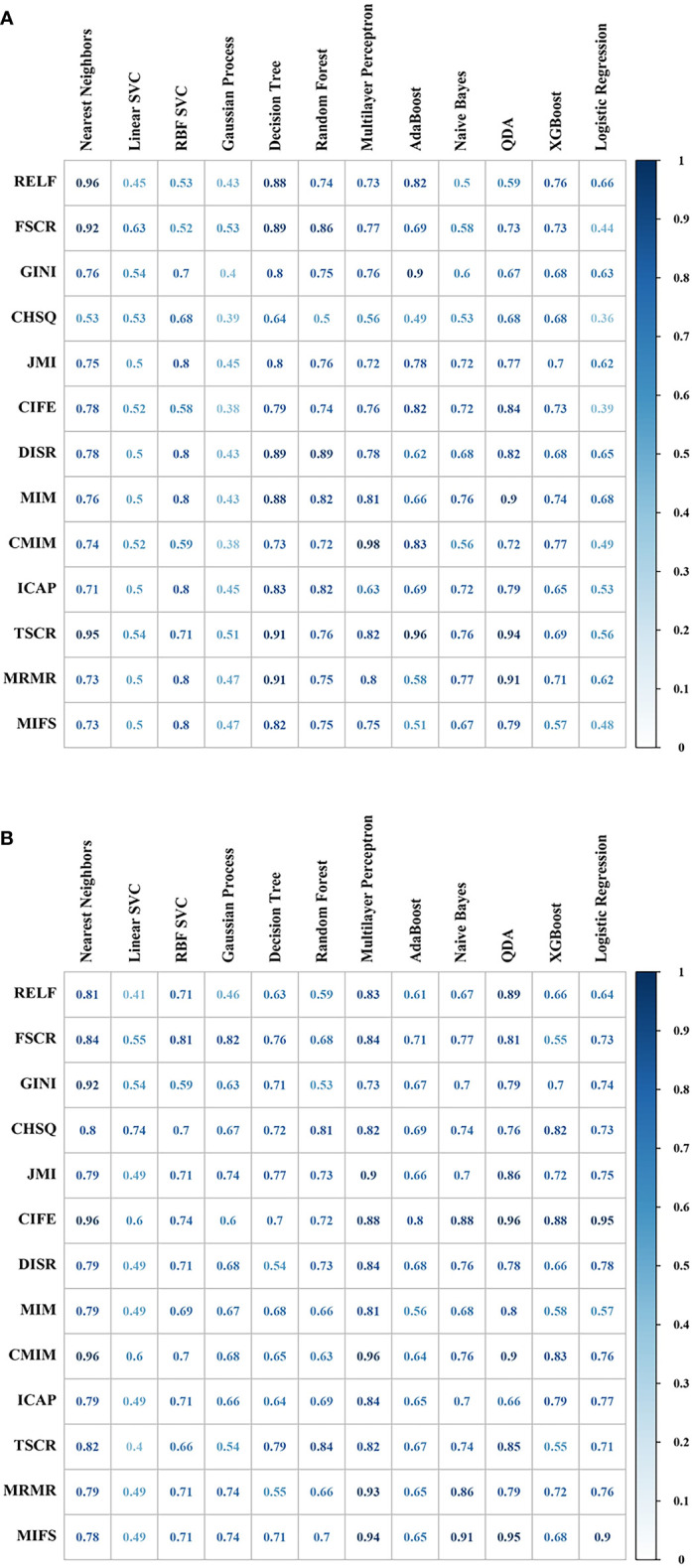
The predictive performance (area under the curve, AUC) of different combinations of feature selection methods (rows) and classification algorithms (columns) were presented in the heatmap. **(A)** Cross-validated AUC values of 156 models with InceptionV3 features. **(B)** Cross-validated AUC values of 156 models with radiomics features.

The optimal model InceptionV3_RELF_ Nearest Neighbors was selected with the AUC value 0.96 and RSD 0.50 among the 1,092 machine-learning models (list of all feature selectors were in **Supplementary Table 3**, and the parameter settings and tuning range of ML methods were presented in **Supplementary Material**). Analysis of the confusion matrix-related classification metrics of InceptionV3_RELF_ Nearest Neighbors showed that the ACC, sensitivity, specificity, precision, and F1 score were 95.24%, 95.00%, 95.50%, 91.67%, and 95.30%, respectively. The illustration of the 10-fold cross-validated AUC for InceptionV3 features was presented in **Figure 4A**. Interestingly, the radiomics models had equal performance. The AUC value of Radiomics_CIFE_Nearest Neighbors, Radiomics_CIFE_QDA, Radiomics_CMIM_Nearest Neighbors, and Radiomics_CMIM_Multilayer Perceptron) was 0.96 in each model, and the RSD was 0.61, 0.67, 0.61, and 0.67. The heatmap of the 10-fold cross-validated AUC concerning radiomics features were presented in **Figure 4B**. Regarding the ML classifiers, the Nearest Neighbors classification outperformed other classifications, with the median AUC 0.79 (IQR 0.75–0.85). **Supplementary Figure 2** reported the mean AUC of the Nearest Neighbors classification.

## Discussion

In this study, by utilizing quantitative image analysis to extract features in conjunction with a ML classifier, we constructed accurate and reproducible models to predict immunotherapy response for advanced NSCLC. Importantly, these efficient models were obtained using cross-validation, and the inputs of the models were robust.

PD-L1 immunohistochemistry (IHC) expression, tumor mutation burden, and tumor-infiltrating lymphocytes have been suggested to predict the response to immunotherapy (24, 25). However, tissue-based biomarkers rely on individual tumor samples from accessible lesions in clinic practice and may not truly reflect the complexity of inter-tumoral heterogeneity. Furthermore, it is difficult to determine the current status of immune profiles from archival samples, as immune-escape mechanisms evolve dynamically during anti-cancer treatment (5, 6).

The main idea of DL is to employ a deep neural network, which provides a unique set of novel tools to improve NSCLC detection (26), characterization (27), survival prediction, and treatment outcome (28). However, compared with statistical ML models, DL models typically required a much larger amount of data to train for optimal results. To overcome the limitations of small datasets, transfer learning patterns (29) facilitate DL models as powerful extractors of useful feature sets.

Radiomic features have been used to predict the benefit of adjuvant chemotherapy, disease risk in early stage lung cancer (30), treatment response to concurrent chemoradiation in locally advanced lung cancer (31), and response to immune checkpoint inhibition in advanced NSCLC (32, 33). Most studies focused on the AUC of predictive models on a given dataset without considering the robustness of imaging features.

Our model is reliable and reproducible, because it uses robust features following the standardization of the model’s input images and can be applied to CT data of various institutions. This model can minimize possible differences between different medical centers, inspection machines, and image reconstruction methods.

The evaluation of the robustness feature is based on the assumption that test–retest images and perturbations do not have consistent bias. We tested the robustness of features against perturbations, such as slice thickness spacing(S), rotation(R), and ROI variation (Seg). Both DL and radiomic features show excellent robustness to S perturbation and have a modest performance to Seg perturbation. The Seg perturbation captured the range of variability that occurred with human inter-observer variability and patient respiratory motion artifact. It is better to underestimate rather than overestimate the ROI when segmenting.

Several major limitations remained in the present study. First, our data were relatively small, and baseline characteristics maybe not in accordance with the population-based dataset. For example, the objective response rate was higher than in the previous study (34, 35). Thirty-two patients chose immunotherapy, because they could not tolerate chemotherapy toxicity rather than disease progression, which partly explained the high efficiency. Second, three consecutive slices of the tumor were sampled for the analysis, and volumetric assessments were not performed. In a previous study, data from a single slice were found to be sufficient for this type of analysis (36). Third, whether our algorithm model for predicting immunotherapy response can be applied to cancer types other than NSCLC is another potential research question to be solved. Fourth, our model lacks external verification. Compared with the DL model, the characteristic stability of radiomics model was higher; however, the prediction capabilities of the DL and radiomics model were comparable. Which model is better requires further verification. Fifth, the factors involved with image features, such as histogram equalization approaches, noise removal methods, and image reconstruction methods, require more in-depth study. Sixth, more study is required to determine whether transfer learning may take the role of the specifically created model for NSCLC due to the heterogeneity between the source and destination databases. In addition, PD-L1 expression data were unavailable for a majority of patients in our cohort. The correlation between PD-L1 expression, which was a clinically validated biomarker of benefit from PD1/PD-L1 blockade, and the instructed model, was not involved in our study.

To the best of our knowledge, this is the first work assessing the robustness of image features in CT imaging of NSCLC patients. In addition, we perform a comparative analysis to select the best machine-learning methods with favorable predictive AUC and stability. Inception V3_RELF_Nearest Neighbors classifiers provided a robust, non-invasive way to identify NSCLC patients who may benefit from immunotherapy. We believe that combining machine-learning methods and radiomics/DL features will improve the AUC in predicting immunotherapy efficacy.

## Data availability statement

The raw data supporting the conclusions of this article will be made available by the authors, without undue reservation.

## Ethics statement

The studies involving human participants were reviewed and approved by the Ethics Committee of Union Hospital. Written informed consent for participation was not required for this study in accordance with the national legislation and the institutional requirements.

## Author contributions

The study was designed by NH, QJ, and QR with the help of the others. QR, PZ, FX, XC, NH, and QJ analyzed and interpreted the data. QR developed a model. QR, QJ, FX, and PZ performed the main computational works. NH, XC, and GW collected surgical data and supported transcriptome analyses. QR, FX, PZ, XC, NH, and QJ collected and analyzed the clinical data. QR, FX, PZ, and QJ performed image analysis and interpretation. GW and NH acquired funding for this study. QR, FX, PZ, NH, and QJ mainly wrote the manuscript, and all authors edited the manuscript. All authors contributed to the article and approved the submitted version.

## Funding

This study was supported by the National Natural Science Foundation of China (No. 82172755) and Wuhan Knowledge Innovation Special (Item Number: 2022020801020532).

## Conflict of interest

The authors declare that the research was conducted in the absence of any commercial or financial relationships that could be construed as a potential conflict of interest.

## Publisher’s note

All claims expressed in this article are solely those of the authors and do not necessarily represent those of their affiliated organizations, or those of the publisher, the editors and the reviewers. Any product that may be evaluated in this article, or claim that may be made by its manufacturer, is not guaranteed or endorsed by the publisher.

## Abbreviations

ICC, Intraclass Correlation Coefficient; NSCLC, non-small cell lung carcinoma; AUC, area under the curve; RSD, relative standard deviation in percentile; PD-1, programmed death 1 receptor; PD-L1, programmed death ligand 1; OS, overall survival; RR, response rates; imRECIST, Immune-Modified Response Evaluation Criteria In Solid Tumors; ROIs, the tumor regions of interest; GLCM, gray-level co-occurrence matrix; GLSZM, gray-level size zone matrix; GLRLM, gray-level run-length matrix; NGTDM, neighborhood gray-tone difference matrix; GLDM, gray-level dependence matrix; CHSQ, chi-square score; RELF, ReliefF; MIM, mutual information maximization; FSCR, Fischer Score; MIFS, mutual information feature selection; GINI, Gini index; ICAP, interaction capping; JMI, joint mutual information; CIFE, conditional infomax feature extraction; CMIM, conditional mutual information maximization; DISR, double input symmetric relevance; MRMR, minimum redundancy maximum relevance; TSCR, t-test score; QDA, quadratic discriminant analysis; SVC, Support Vector Classifiers; RBF, radial basis function; ACC, accuracy; DL, deep learning.
