# Supplementary material for: Assessing the robustness of radiomics/deep learning approach in the identification of efficacy of anti–PD-1 treatment in advanced or metastatic non-small cell lung carcinoma patients
Source: Front Oncol. 2022 Aug 5;12:952749. doi: 10.3389/fonc.2022.952749 (PMC9390967; doi:10.3389/fonc.2022.952749)
Supplement: Supplementary file 1 [file DataSheet_1.docx]

Supplementary Material

# Supplementary Data

**1.1 Response assessment**

The thoracic radiologists identified and measured two target lesions per organ to a maximum of five lesions on baseline and post therapy scans for every patient. The target lesions observed on two consecutive CT scans were evaluated by Immune-Modified Response Evaluation Criteria In Solid Tumors (imRECIST) (1). The sum of diameters of the target lesions (longest for non-nodal lesions and the short axis for the nodal region) were compared for baseline, and post therapy scans. Patients who had progressive disease after treatment (PD, defined as at least 20% increase in the sum of the longest diameter of target lesions) were classified as “nonresponders,” and patients who had radiographic response, including complete response (the disappearance of all the lesions), partial response (≥30% decrease in the sum of the longest diameters of target lesions) or stable disease (neither PR or PD) were classified as “responders”.

**1.2 Machine learning methods**

In the following section, we list the parameter settings and tuning range of the ML mthods.

• Nearest Neighbors (2): Number of neighbors varied in the range from three to 21, in steps of 3

• Support vector classifier (3), with linear kernel, penalty term C = {0.25, 0.5, 1, 2, 4}

• Support vector classifier (3), with RBF kernel, penalty term C = {0.25, 0.5, 1, 2, 4}, and gamma = {'scale', 'auto', 0.01, 0.1, 1, 10, 100}

• Gaussian process classifier (4): kernel set to 1.0 * RBF (1.0)

• Decision tree (5): Maximum depth tuned in the range {5, 10, 15, 20}

• Random forests (6): 100 trees and maximum number of features set to 'auto'

• Multilayer perceptron (7): Maximum iterations set to 5000, alpha = {0.0001, 0.001, 0.01, 0.1, 1, 10}

• AdaBoost (8, 9): Default settings

• Naïve Bayes (10): Gaussian naïve Bayes classifier, without priors, var_smoothing at default value (1e-9)

• Quadratic discriminant analysis (QDA) (11): No priors, covariate estimate regularization at default 0.

• XGBoost (12): No instance weighting

• Logistic regression (13), default settings (penalty=’l2’, dual=False, tol=0.0001, C=1.0, fit_intercept=True, intercept_scaling=1, class_weight=None, random_state=None, solver=’warn’, max_iter=100, multi_class=’warn’, verbose=0, warm_start=False, l1_ratio=None)

**REFERENCE**

1. Hodi FS, Ballinger M, Lyons B, Soria JC, Nishino M, Tabernero J, et al. Immune-Modified Response Evaluation Criteria In Solid Tumors (imRECIST): Refining Guidelines to Assess the Clinical Benefit of Cancer Immunotherapy. J Clin Oncol. 2018;36(9):850-8

2. Cover T, Hart P. Nearest neighbor pattern classification. IEEE Trans Inf Theory. 1967;13(1):21-27. doi:10.1109/TIT.1967.1053964

3. Platt JC, Platt JC. Probabilistic Outputs for Support Vector Machines and Comparisons to Regularized Likelihood Methods. Adv LARGE MARGIN Classif. 1999:61--74. http://citeseer.ist.psu.edu/viewdoc/summary?doi=10.1.1.41.1639. Accessed December 18, 2019.

4. Rasmussen CE, Williams CKI. Gaussian Processes for Machine Learning. MIT Press; 2006. http://www.gaussianprocess.org/gpml/. Accessed December 18, 2019.

5. Gordon AD, Breiman L, Friedman JH, Olshen RA, Stone CJ. Classification and Regression Trees. Biometrics. 1984;40(3):874. doi:10.2307/2530946

6. Breiman L. Random Forests. Mach Learn. 2001;45(1):5-32. doi:10.1023/A:1010933404324

7.Hinton GE. Connectionist Learning Procedures. Mach Learn. January 1990:555-610. doi:10.1016/B978-0-08-051055-2.50029-8

8. Hastie T, Rosset S, Zhu J, Zou H. Multi-class AdaBoost. Stat Interface. 2009;2(3):349-360. doi:10.4310/SII. 2009.v2. n3. a8

9. Freund Y, Schapire RE. A Decision-Theoretic Generalization of On-Line Learning and an Application to Boosting. J Comput Syst Sci. 1997;55(1):119-139. doi:10.1006/JCSS.1997.1504

10. Chan TF, Golub GH, LeVeque RJ. Updating Formulae and a Pairwise Algorithm for Computing Sample Variances. In: COMPSTAT 1982 5th Symposium Held at Toulouse 1982. Heidelberg: Physica-Verlag HD; 1982:30-41. doi:10.1007/978-3-642-51461-6_3

11. Ledoit O, Wolf M. Honey, I Shrunk the Sample Covariance Matrix. J Portf Manag. 2004;30(4):110-119. doi:10.3905/jpm.2004.110

12. Chen T, Guestrin C. XGBoost: A Scalable Tree Boosting System. March 2016. doi:10.1145/2939672.2939785

13. Verhulst PF. La loi d’accroissement de la population. 1845.

# Supplementary Figures and Tables

## Supplementary Figures

**
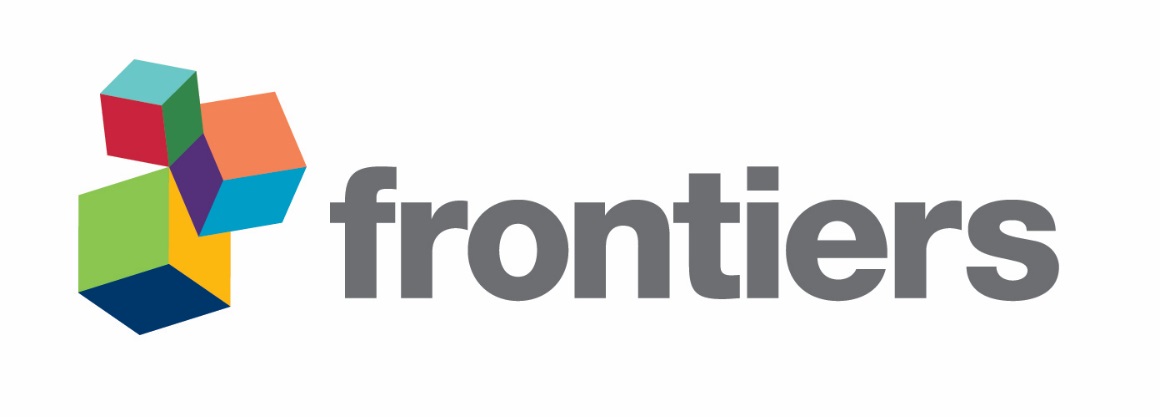
**

**Supplementary Figure 1.** Variability of feature robustness within feature extractors for the tested perturbations.

**Supplementary Figure 2.** The predictive performance (AUC) of 91 models with k Nearest Neighbors classifier.

## Supplementary Tables

Table S1: The ICC was listed as median (IQR) across regarding image perturbation.

| **Feature exactor** | **Slice thickness** | **Rotate** | **Variation of ROI** |
| --- | --- | --- | --- |
| InceptionResNetV2 | 0.90(0.87-0.92) | 0.87(0.81-0.91) | 0.82(0.76-0.86) |
| InceptionV3 | 0.89(0.86-0.91) | 0.83(0.77-0.88) | 0.78(0.72-0.84) |
| Resnet50 | 0.92(0.89-0.95) | 0.85(0.75-0.91) | 0.85(0.78-0.90) |
| VGG16 | 0.93(0.88-0.96) | 0.83(0.68-0.90) | 0.89(0.80-0.94) |
| VGG19 | 0.93(0.89-0.96) | 0.85(0.71-0.91) | 0.88(0.80-0.94) |
| Xception | 0.88(0.80-0.92) | 0.81(0.65-0.88) | 0.84(0.74-0.89) |
| Radiomics | 0.94(0.89-0.96) | 0.94(0.89-0.97) | 0.97(0.93-0.99) |

Table S2: The ICC was listed as median (IQR) across regarding test-retesting.

| **Feature exactors** | **ICC** |
| --- | --- |
| InceptionResNetV2 | 0.43(0.24-0.55) |
| InceptionV3 | 0.43(0.25-0.59) |
| Resnet50 | 0.50(0.28-0.65) |
| VGG16 | 0.45(0.15-0.66) |
| VGG19 | 0.57(0.24-0.79) |
| Xception | 0.42(0.06-0.64) |
| Radiomics | 0.67(0.45-0.77) |

Table S3: List of all feature selectors.

| **Name** | **Abbreviation** |
| --- | --- |
| ReliefF | RELF |
| Fischer Score | FSCR |
| Gini index | GINI |
| Chi-square score | CHSQ |
| Joint mutual information | JMI |
| Conditional infomax feature extraction | CIFE |
| Double input symmetric relevance | DISR |
| Mutual information maximization | MIM |
| Conditional mutual information maximization | CMIM |
| Interaction capping | ICAP |
| T-test score (only for binary classification) | TSCR |
| Minimum redundancy maximum relevance | MRMR |
| Mutual information feature selection | MIFS |
