# Supplementary figures and images for: Assessing the robustness of radiomics/deep learning approach in the identification of efficacy of anti–PD-1 treatment in advanced or metastatic non-small cell lung carcinoma patients
Source: Front Oncol. 2022 Aug 5;12:952749. doi: 10.3389/fonc.2022.952749 (PMC9390967; doi:10.3389/fonc.2022.952749)

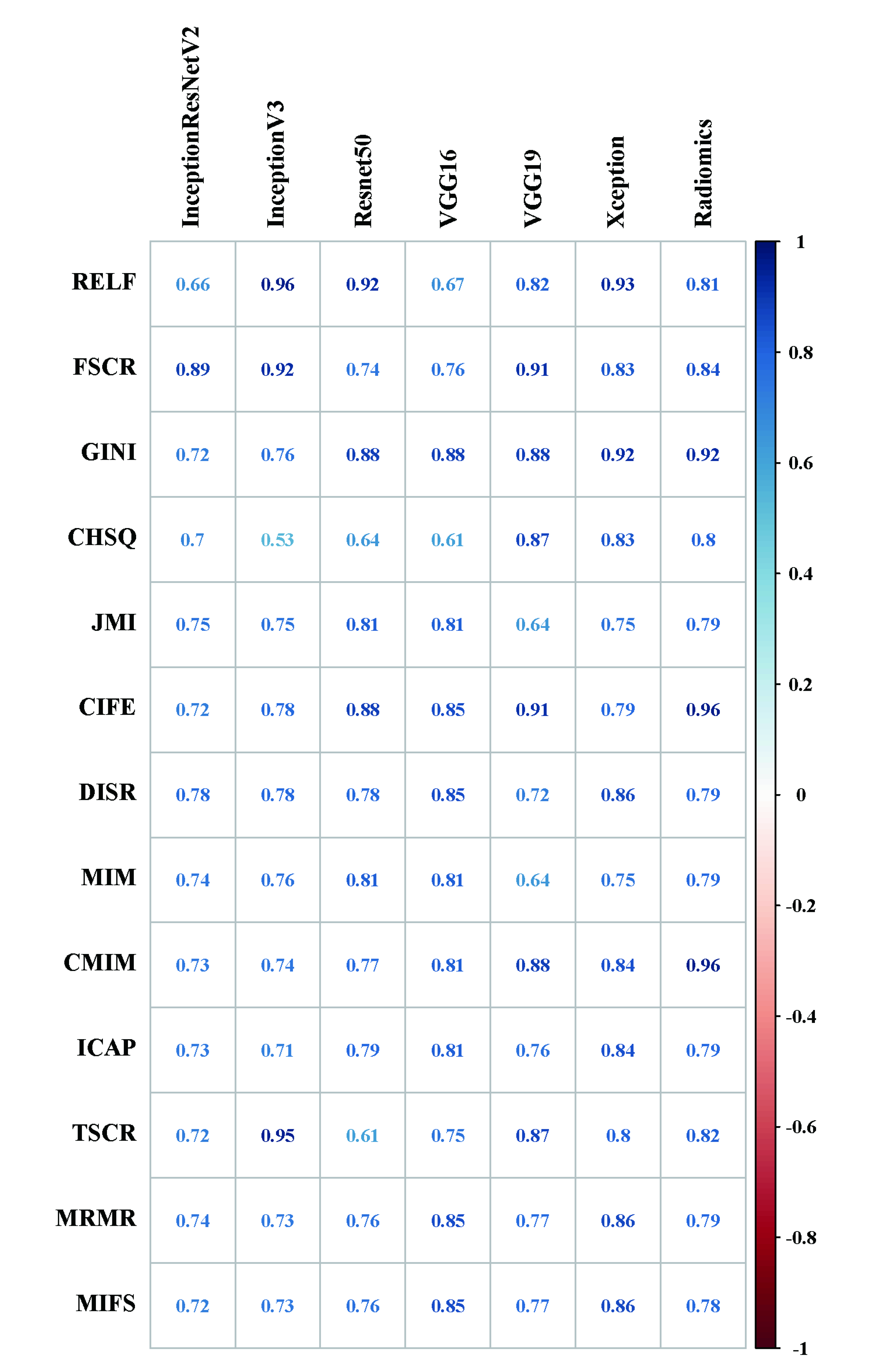

Supplement: Supplementary file 3 [file Image_2.tif]
